# CUSUM Analysis as a Predictive Tool for the Learning Curve in Left Bundle Branch Area Pacing

**DOI:** 10.3390/jcm15010335

**Published:** 2026-01-01

**Authors:** Ionuț Tudorancea, Mihai Ștefan Cristian Haba, Mircea Cristian Placinta, Irene Paula Popa, Emil Gabriel Calistrat, Alexandru Guila, Daniela Crișu, Viviana Onofrei, Dragomir Nicolae Șerban, Ionela Lăcrămioara Șerban, Radu Iliescu, Irina Iuliana Costache Enache

**Affiliations:** 1 Grigore T. Popa University of Medicine and Pharmacy, 700115 Iasi, Romania; ionut.tudorancea@umfiasi.ro (I.T.); irene-paula_popa@umfiasi.ro (I.P.P.); irina.costache@umfiasi.ro (I.I.C.E.); 2 Cardiology Clinic, “Saint Spiridon” County Clinical Emergency Hospital, 700111 Iasi, Romania; 3 Transcend Research Centre, 2-4 General Henri Mathias Berthelot Street, 700483 Iaşi, Romania

**Keywords:** left bundle branch pacing, conduction system pacing, cumulative sum (CUSUM) analysis, learning curve, operator training, cardiac resynchronization therapy

## Abstract

**Background/Objectives**: Left bundle branch area pacing (LBBAP) has rapidly emerged as a physiological alternative to conventional pacing. However, the learning curve for new operators remains poorly characterized. We aimed to describe the learning curve of LBBAP using cumulative sum (CUSUM) analysis in a single-center, single-operator setting. **Methods**: We conducted a retrospective analysis of 108 consecutive LBBAP attempts performed by a first-time operator with no prior conduction system pacing experience. Fluoroscopy time was selected as the primary performance metric. A predefined procedural target (≤6 min for non-CRT and ≤9 min for CRT implants) was used to classify each case as a success or failure. CUSUM plots were generated to identify the inflection point marking proficiency acquisition. **Results**: In non-CRT cases (*n* = 95), fluoroscopy time progressively decreased, and the CUSUM curve showed a distinct inflection after approximately 65 cases, indicating the attainment of procedural proficiency. In CRT implants (*n* = 9), acceptable fluoroscopy exposure was achieved soon after the non-CRT proficiency threshold was reached. Electrical performance and safety outcomes remained stable throughout the learning process. **Conclusions**: CUSUM provides a clear, objective, and real-time measure of operator progression during the initial learning phase of LBBAP. The technique reliably identified the proficiency transition and can be readily implemented for procedural training, credentialing, and competency monitoring in centers adopting LBBAP.

## 1. Introduction

Cardiac conduction disorders are a major cause of morbidity, for which permanent pacemaker implantation remains the standard therapy in patients with symptomatic bradycardia. Traditionally, right ventricular apical pacing (RVAP) has been the standard approach; however, the electrical and mechanical dyssynchrony associated with RVAP increases the risk of atrial fibrillation, heart failure, and overall mortality [1,2,3,4,5]. Alternative pacing sites, such as the right ventricular septum and outflow tract, have not consistently yielded superior long-term outcomes [6,7].

Left bundle branch pacing (LBBP) has recently emerged as a promising physiological pacing technique. By directly stimulating the left conduction system distal to the His bundle, LBBP enables synchronous ventricular activation. Multiple studies have demonstrated its efficacy in both bradycardia and resynchronization indications, with improved electrical synchrony and favorable clinical outcomes compared with conventional pacing [8,9,10,11,12,13,14].

Despite its increasing adoption, evidence on the learning curve for LBBP remains scarce. Although several studies have recently explored this topic, such as the learning-curve analyses [14,15,16,17], most investigations were performed by experienced operators or in high-volume centers. Therefore, a clear unmet need persists for real-world data describing the performance trajectory of a first-time operator initiating a de novo conduction-system pacing program.

While previous studies have described the evolution of operator experience with LBBP, they have largely relied on descriptive or linear trend analyses that offer limited predictive insight. In contrast, the cumulative sum (CUSUM) technique—widely adopted in surgical and interventional disciplines—provides a dynamic, real-time, case-by-case measure of performance improvement and can objectively predict the point of procedural proficiency. Applying this methodology to LBBAP introduces a quantitative framework for assessing learning efficiency and for anticipating when proficiency has been achieved [18,19].

The present study aimed to evaluate the procedural and electrical outcomes of LBBAP performed by a single operator with no prior experience in conduction system pacing, and to characterize the learning curve using fluoroscopy time and radiation exposure as objective performance metrics. In addition, this study sought to demonstrate the utility of CUSUM analysis as a predictive tool capable of identifying the transition from learning to proficiency in real time, offering a novel framework for training and quality assessment in conduction system pacing.

## 2. Materials and Methods

### 2.1. Study Design and Setting

We conducted a retrospective, single-center observational study including 108 consecutive patients who underwent left bundle branch area pacing (LBBAP) implantation between October 2024 and July 2025 at “St. Spiridon” Emergency Clinical Hospital, Iasi, Romania. All procedures were performed by the same operator who had extensive prior experience in conventional cardiac device implantation. This background ensured a high level of proficiency with transvenous lead handling and device-implantation workflows prior to initiating the LBBAP program. This design of our study not only allowed a clear evaluation of the procedural learning curve, but also reflected institutional practice, as a single cardiologist was accredited and authorized to perform conduction system pacing implantations.

### 2.2. Patient Selection

The conduction system pacing program was initially launched for bradycardia indications only, and CRT indications were introduced later as operator experience increased. During the first 20 cases, patients with near-normal anatomy were selected. Although persistent left superior vena cava does not preclude LBBAP—as demonstrated in several case reports [20,21]—it represents a technically challenging anatomical variant and was therefore excluded early in the learning program. More complex anatomies (e.g., enlarged right atria, enlarged right ventricle, severe tricuspid regurgitation, interventricular septum hypertrophy) were gradually included, and after the 27th case, indications were expanded to patients eligible for cardiac resynchronization therapy (CRT). Notably, procedural complexity was purposely staged: the initial cases involved patients with standard anatomy, while more complex anatomies (e.g., enlarged right atrium or ventricle, severe tricuspid regurgitation, hypertrophied septum) and CRT indications were introduced only after initial experience accumulated. Thus, the case mix evolved gradually and did not systemically bias early versus late fluoroscopy performance.

Comorbidities were assessed based on clinical records. Chronic kidney disease (CKD) was defined as an estimated glomerular filtration rate < 60 mL/min/1.73 m^2^ or a documented diagnosis of CKD stage ≥ 3. Atrial fibrillation (AF) was defined as paroxysmal, persistent, or permanent AF documented by ECG or Holter monitoring.

### 2.3. Implantation Technique

Implantation was performed using a Medtronic 3830 lumenless pacing lead delivered via C315 His or C304 sheaths, with venous access through the cephalic or subclavian vein. Septal engagement was guided fluoroscopically using RAO 30° and LAO 40° projections. A backup RV apical lead was positioned initially and removed after stable LBB capture. For CRT-D patients, a defibrillator lead was implanted in the apex of the right ventricle.

### 2.4. Management of Complications

Potential complications (septal perforation, lead instability, coronary injury, pericardial effusion) were actively monitored. Lead repositioning and backup RV lead retention were employed as safety measures when necessary.

### 2.5. Electrical Measurements

**Figure 1:**
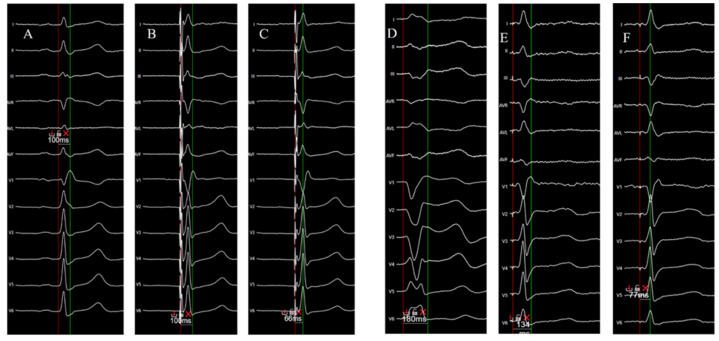
Twelve-lead ECG examples illustrating measurement of native and paced QRS duration and V6 R-wave peak time (V6-RWPT) in two representative patients. Panels (**A**–**C**) depict a bradycardia patient treated with a conventional pacemaker (non-CRT). (**A**): native QRS duration (red vertical line marks QRS onset), measured at 100 ms. (**B**): paced QRS duration, 100 ms. (**C**): paced V6-RWPT = 66 ms, measured from QRS onset (red line) to the R-wave peak (green line), consistent with left bundle branch capture. Panels (**D**–**F**) depict a patient with a cardiac resynchronization therapy indication. (**D**): baseline wide QRS (180 ms, left bundle branch block morphology). (**E**): paced/narrowed QRS after implantation (134 ms). (**F**): paced V6-RWPT = 77 ms, measured from the red (onset) to the green (R-peak) vertical marker. Together, the shortening of QRS duration and the short V6-RWPT support physiological ventricular activation via the left conduction system.

Electrical data were recorded using the EP Tracer system (Schwartzer Cardiotek). The procedural endpoint was achievement of left bundle branch area capture (LBBAP), confirmed when one or more EHRA-defined criteria were present: transition between non-selective and selective capture during threshold testing; paced V6-RWPT compatible with LBB capture; r’/R’ morphology in V1; a narrow paced QRS with preserved morphology; or current of injury (COI). Deep septal pacing (DSP) morphology was observed in 15.4% of cases (16/104), consistent with the expected morphological spectrum of LBBAP. Pacing outcomes were classified into two categories: EHRA-confirmed left bundle branch capture, and non–LBB capture septal pacing, which included both deep septal pacing (DSP) and left ventricular septal pacing (LVSP). LVSP was grouped with DSP because both represent septal pacing without fulfillment of LBB capture criteria. This classification was used exclusively for reporting capture outcomes; all cases were retained in the fluoroscopy-based learning curve analysis. Pacing thresholds were measured using a pulse duration of 0.4 ms. An example of 12-lead ECG illustrating measurements of native and paced QRS duration and pV6-RWPT in 2 non-CRT and CRT patients is depicted in Figure 1.

### 2.6. Endpoints and Statistical Analysis

The primary endpoints were fluoroscopy time (seconds) and radiation dose (mGy). Patients were analyzed in chronological order based on the actual date of implantation. For descriptive analysis, a 10-case moving average was applied. Linear regression tested temporal trends.

To objectively identify proficiency, cumulative sum (CUSUM) analysis was applied separately to non-CRT (DDD and VVI) and CRT implants. The fluoroscopy time thresholds used for the CUSUM analysis were set a priori based on published efficiency data and the real-world workflow in our center. Prior studies have reported end-procedural fluoroscopy times of ~4–5 min for ventricular lead placement [15] and ~8–12 min for complete single- and dual-chamber implants in experienced centers [16,17,22]. We therefore pre-specified conservative targets of 6 min (360 s) for non-CRT and 9 min (540 s) for CRT procedures. These thresholds were designed to represent achievable and clinically meaningful total procedural fluoroscopy time, including the deployment and removal of backup right ventricular leads. They lie within the reported range for complete implants and were chosen to provide a realistic performance goal for an operator early in the learning phase. Defining these cutoffs before analysis ensured a transparent and methodologically valid success/failure classification for CUSUM. CUSUM charts were constructed separately for non-CRT and CRT implants by assigning each case a value of +1 (failure) when fluoroscopy exceeded the target and –1 (success) when it met or fell below the target and plotting the cumulative sum over time. The CUSUM inflection point was used to identify the transition from learning to proficiency. Descriptive statistics were performed using IBM SPSS Statistics, version 26.0 (IBM Corp., Armonk, NY, USA), whereas CUSUM analysis was conducted in R version 4.3.2 (R Foundation for Statistical Computing, Vienna, Austria) using the qcc package.

### 2.7. Ethical Considerations

All patients provided written informed consent. The study was approved by the local ethics committee and conducted in accordance with the Declaration of Helsinki.

## 3. Results

Between October 2024 and July 2025, 108 patients attempted LBB pacing implantation at our institution. Four patients were excluded due to anatomical challenges that prevented stable lead positioning, leaving 104 patients in the final analysis. The chronological distribution of implantations revealed a progressive increase in procedural complexity, beginning with simpler DDD or VVI pacemaker systems and later expanding to CRT and CRT-D procedures as operator confidence improved.

The baseline demographic and clinical characteristics of the study population are summarized in Table 1. The mean age was 71.9 ± 7.3 years, with 65.4% male. The most frequent pacing indication was atrioventricular block (74 patients), followed by sick sinus syndrome (21 patients) and CRT indication (9 patients). Device types included 82 dual-chamber pacemakers (DDD), 13 single-chamber pacemakers (VVI), and 9 cardiac resynchronization therapy (CRT) systems. Comorbidities were frequent, with dyslipidemia, diabetes mellitus, and chronic kidney disease among the most prevalent.

As summarized in Table 2, electrical parameters at implantation and at 1 and 3 months follow-up were favorable, with consistently low thresholds, high R-wave amplitudes, and stable lead impedances. At implantation, the mean pacing threshold was 0.82 ± 0.22 V, with an impedance of 794 ± 170 Ω and R-wave sensing amplitude of 17.8 ± 6.8 mV. In non-CRT patients, the paced R-wave peak time in lead V6 (pRWPT6) averaged 70.7 ± 8.2 msec, with more than 90% achieving the target of ≤75 msec. Conduction system capture was assessed using EHRA criteria. When present, it was supported by findings such as a paced QRS with preserved repolarization morphology, the absence of terminal conduction delay, a paced V6-RWPT within the physiologic range, or an r’/R’ pattern in lead V1. As expected in LBBAP, these features were not uniformly present in all patients, and using EHRA-defined electrophysiological criteria, successful left bundle branch capture was achieved in 88 of 104 procedures (84.6%), while the remaining 16 cases (15.4%) demonstrated DSP and LVSP morphology. In CRT patients, at baseline, the intrinsic QRS duration was prolonged, with a mean value of 175.9 ± 10.2 msec. After LBBA pacing, the paced QRS duration significantly decreased to 138.6 ± 10.4 msec, resulting in an average QRS narrowing of 37.3 ± 11.9 msec. The pRWPT6 interval showed a mean value of 83.7 ± 8.3 msec. At 1 and 3 months follow-up, pacing thresholds declined to 0.42 ± 0.20 V and 0.47 ± 0.18 V, respectively; impedance decreased to 527 ± 83 Ω and 510.53 ± 81.18 Ω, and sensing amplitudes remained robust, highlighting the long-term stability of lead performance.

**Figure 2:**
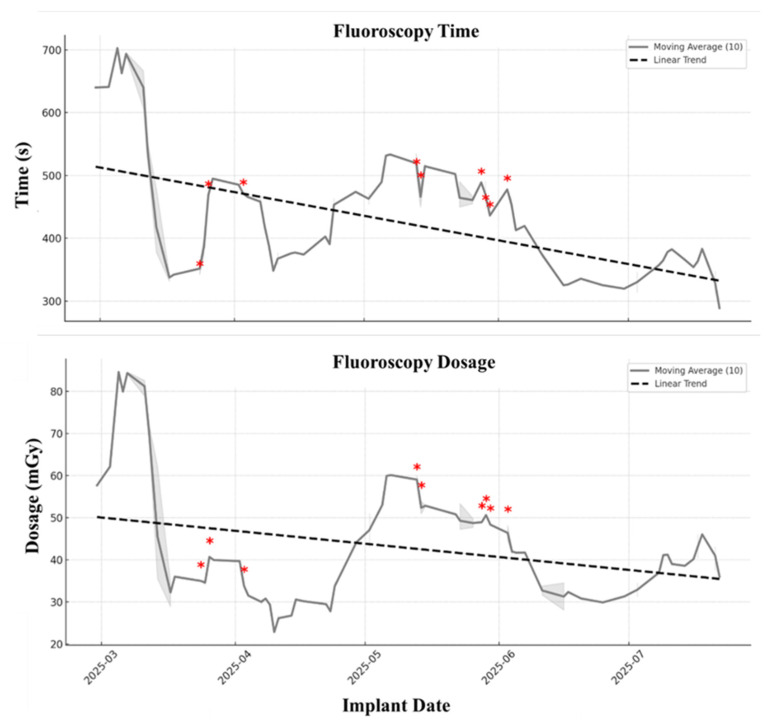
Smoothed learning curves of fluoroscopy exposure during left bundle branch pacing implantations. (**Upper Panel**): fluoroscopy time (seconds) plotted chronologically by implant date. The solid grey line represents the 10-case moving average, while the dashed black line indicates the linear regression trend. A progressive decline is observed across the study period, consistent with improved procedural efficiency. (**Lower Panel**): radiation dose (mGy) plotted in the same manner. Although inter-case variability was present, the overall trend showed a gradual decrease in exposure with accumulating operator experience. Red asterisks mark CRT implants.

Procedural outcomes demonstrated a clear learning effect. Chronological analysis showed a steady decline in fluoroscopy time, which was statistically significant by linear regression (slope −1.26 s/day, R^2^ = 0.109, *p* = 0.0006). The radiation dose exhibited a downward trend that did not reach statistical significance (slope –0.10 mGy/day, *p* = 0.109). These temporal patterns are depicted in Figure 2, which illustrates the 10-case moving averages of fluoroscopy time and radiation dose, respectively.

Multiple linear regression was used to assess the association between operator experience and clinical variables with Exposure Time and Exposure Dosage. Predictors included procedure number, device complexity coded by number of implanted leads (CRT/CRT-D = 3, DDD = 2, VVI = 1), right-atrial size, interventricular septum thickness, and pulmonary hypertension. For Exposure Time, the model explained 16.7% of the variance (R^2^ = 0.167, overall model *p* = 0.0027). Procedure number was a significant negative predictor (β = −2.33, *p* = 0.0015), indicating reduced fluoroscopy duration and shorter exposure with increasing experience. Right-atrial size showed a non-significant trend toward longer exposure (β = +6.16, *p* = 0.071), while device complexity, septum thickness, and pulmonary hypertension were non-significant. For Exposure Dosage, the model explained 11.3% of the variance (R^2^ = 0.113, overall model *p* = 0.036). Dose tended to decrease with experience (β = −0.23, *p* = 0.065), and larger right atria were associated with higher doses (β = +1.51, *p* = 0.012). Other predictors were not significant. These findings indicate that the reduction in fluoroscopy time primarily reflected operator learning rather than differences in anatomical or procedural complexity.

**Figure 3:**
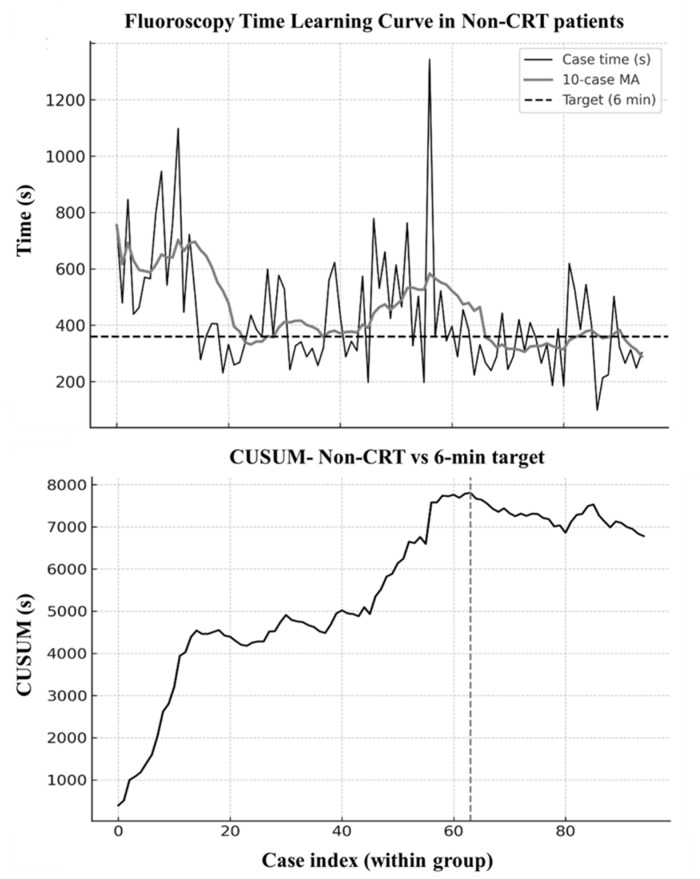
Fluoroscopy time learning curve for non-CRT procedures (DDD and VVI). (**Upper Panel**): individual fluoroscopy times (black) plotted sequentially with a 10-case moving average (gray). The dashed horizontal line represents the predefined target of 6 min (360 s). After approximately the 65th case, fluoroscopy times stabilize below the target. (**Lower Panel**): cumulative sum (CUSUM) analysis for the same cases. The curve rises during the early learning phase and reaches its maximum inflection at case ~65, indicating the transition to procedural proficiency. Thereafter, the CUSUM curve flattens and begins to decline, reflecting sustained achievement of the performance target.

To further delineate the learning curve, CUSUM analysis was performed using prespecified targets of 6 min for non-CRT procedures and 9 min for CRT procedures, based on prior literature benchmarks. In the non-CRT subgroup (95 patients), the CUSUM curve (Figure 3) identified an inflection point after approximately 65 consecutive cases (26 May 2025). Beyond this threshold, fluoroscopy times plateaued around 5 min, with a post-change median of 309 s, and nearly 68% of cases met the ≤6 min target. The corresponding raw fluoroscopy times with a 10-case moving average are shown in Figure 3.

**Figure 4:**
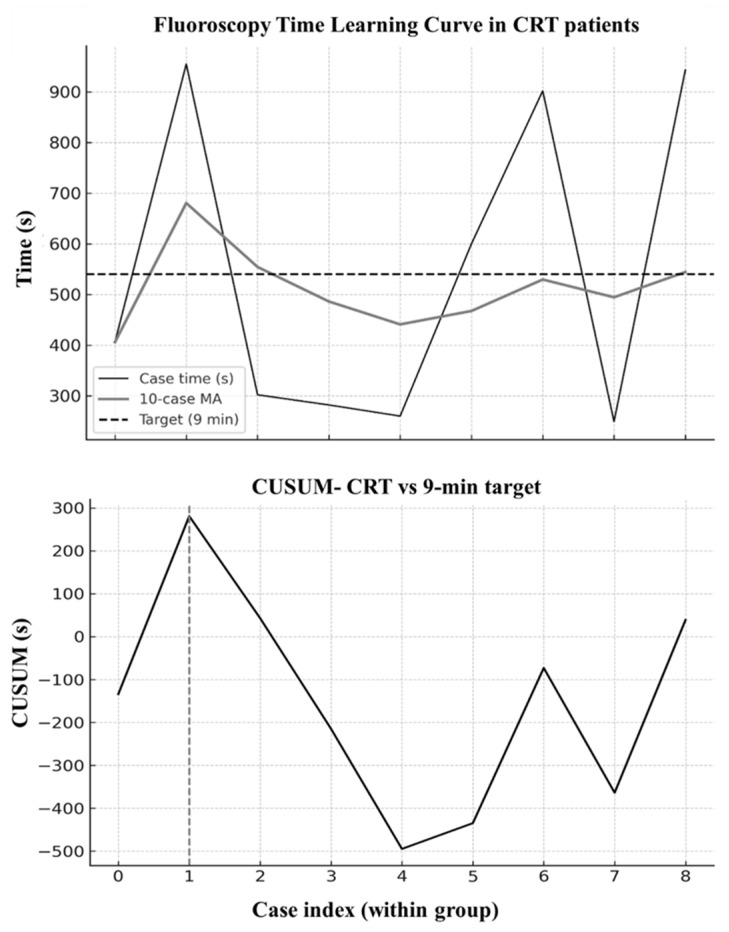
Fluoroscopy time learning curve for CRT procedures. (**Upper Panel**): individual fluoroscopy times (black) with a 10-case moving average (gray). The dashed horizontal line represents the predefined target of 9 min (540 s). Despite the limited number of CRT implants (*n* = 9), most cases remained well below the target, with median values close to 5 min. (**Lower Panel**): cumulative sum (CUSUM) analysis relative to the 9-min target. The curve reaches its maximum inflection at the second case, suggesting early attainment of acceptable procedural performance. Given the small sample size, these results should be interpreted cautiously.

In the CRT subgroup, the CUSUM curve (Figure 4) indicated a performance change point as early as the second CRT case (26 March 2025). Thereafter, fluoroscopy times remained favorable, with a post-change median of 302 s, and 57% of CRT procedures meeting the ≤9-min target. The chronological time series with a moving average is shown in Figure 4. Throughout the study period, procedural safety was preserved. No major complications occurred. Minor complications consisted of pocket hematoma not requiring intervention in 6 patients (5.8%), all occurring in the context of oral anticoagulant therapy, and transient localized pruritus at the device pocket in 2 patients (1.9%), both resolving spontaneously. No cases of lead dislodgement, septal perforation, pneumothorax, pericardial effusion, coronary injury, infection, threshold rise, or loss of conduction-system capture were documented.

Taken together, these findings suggest that while electrical stability was achieved early and maintained consistently, procedural efficiency required repeated exposure before plateauing, with proficiency for non-CRT cases achieved after approximately 65 procedures.

## 4. Discussion

Our study provides a comprehensive characterization of the learning curve for left bundle branch area pacing (LBBAP) initiated by a first-time operator in a real-world setting. Statistical analyses were performed using simple correlations and multivariable linear regression and consistently showed that procedural efficiency was driven predominantly by operator experience. In the multivariable model, each additional procedure was associated with a reduction of ~2.33 s in fluoroscopy time. By contrast, right atrial size, interventricular septal thickness, pulmonary hypertension, and device configuration (VVI/DDD/CRT) were not significant predictors; for radiation dose, experience showed only a non-significant downward trend. These findings indicate that improvements in efficiency primarily reflect progressive skill acquisition rather than patient- or anatomy-related factors.

Given that operator experience emerged as the principal determinant of efficiency, we next sought a quantitative approach to identify a performance threshold and dynamically track proficiency. The chronological analysis, supported by both moving average smoothing (Figure 2) and CUSUM methodology (Figure 3 and Figure 4), offers a detailed depiction of how procedural efficiency evolved over time. Importantly, although fluoroscopy times were longer during the early phase, procedural endpoints were consistently achieved, and conduction-system capture remained frequent throughout the series, with EHRA-defined left bundle branch capture occurring in 84.6% of cases, while deep septal pacing represented the remaining cases. To better clarify the interpretation of pacing outcomes, we distinguished that cases exhibiting LVSP morphology were included within the DSP category, consistent with contemporary EHRA definitions. LVSP and DSP share the same implantation mechanics, fluoroscopic workflow, and septal target depth; the distinction becomes apparent only during post-deployment electrophysiological assessment. Therefore, LVSP was grouped with DSP for reporting non-LBB capture outcomes, while all learning-curve analyses incorporated the full LBBAP cohort because fluoroscopy time reflects procedural workflow rather than the eventual capture subtype. The gradual decline in fluoroscopy times, concluding in a plateau after approximately 65 non-CRT cases, demonstrates the operator’s transition from the learning phase to procedural proficiency, such as sheath navigation, septal engagement, and recognition of electrocardiographic markers of conduction system capture. The CUSUM inflection point provided an objective confirmation of this transition from learning to proficiency. Although all CRT procedures in our cohort fulfilled EHRA criteria for left bundle branch area capture, the small sample size (*n* = 9) does not allow characterization of a CRT-specific learning curve. These observations should therefore be interpreted as preliminary feasibility rather than evidence of CRT procedural proficiency. Our findings support the incorporation of structured LBBAP training pathways in which novice operators begin with anatomically favorable non-CRT cases and progress in complexity under guided performance monitoring.

This study shows that operator experience is the dominant factor shaping the learning curve of left bundle branch area pacing (LBBAP), with procedural efficiency steadily improving as experience accumulates, while electrical performance and safety remain stable throughout. Other clinical or anatomical variables contributed minimally, underscoring that the observed gains primarily reflect true skill acquisition rather than differences in case complexity. Although procedural complexity increased after the introduction of CRT implants and more challenging anatomies, multivariable analysis showed that neither device type nor anatomical characteristics (right atrial size, septal thickness, pulmonary hypertension) significantly influenced fluoroscopy time. Thus, the improvement in efficiency is best explained by progressive operator proficiency rather than favorable case selection.

Building on this finding, we applied CUSUM analysis to define the point at which the operator transitioned from the learning phase to consistent proficiency. Chronological trends and CUSUM curves demonstrated a clear shift from longer, variable fluoroscopy times early in the series to a stable plateau once core technical competencies—such as sheath manipulation, septal engagement, and recognition of conduction-system capture—had been mastered. Electrical outcomes remained reliable from the outset, and no major complications occurred, indicating that LBBAP can be introduced safely even during the operator’s early learning phase. Even though the number of CRT procedures (*n* = 9) was limited, fluoroscopy targets were reached early in this subgroup, suggesting that once fundamental LBBAP skills are established, additional procedural complexity does not markedly prolong exposure. However, due to the small sample size, these results should be interpreted as feasibility observations only. The study was not powered to define a CRT-specific learning curve, and no conclusions about CRT proficiency were drawn.

Collectively, these findings support structured training pathways in which novice operators begin with straightforward non-CRT cases and advance gradually under guided performance monitoring.

Our findings complement and extend prior descriptions of the LBBAP learning curve. Our results can be positioned within the spectrum of published reports. Yu et al. [22] described a plateau after only 25 cases in novice operators, with early fluoroscopy times averaging 15 min. Clark et al. [17] observed proficiency after 10–15 cases among operators already experienced with device implantation, with fluoroscopy declining from 12 to 8 min. Heckman et al. [16], in contrast, found a longer trajectory with stabilization after 40–60 implantations and fluoroscopy times of ~12 min in later cases. Finally, Wang and colleagues [15], in a large series of 406 patients, reported a three-phase learning curve, with final stabilization after ~150 cases and fluoroscopy times of 4–5 min, though only ventricular lead fluoroscopy was measured.

In our single-operator de novo program, the CUSUM-defined proficiency threshold for non-CRT LBBAP was reached after approximately 65 cases, with fluoroscopy times subsequently stabilizing around 5 min. This threshold is higher than the 10–25 cases reported for experienced operators but falls within the broader 40–60 case range described by Heckman et al. Several factors likely contribute to this difference: unlike Yu [19] or Wang [17], we measured total procedural fluoroscopy time, including here implantation and extraction of the back-up RV lead, or implantation of atrial and defibrillator leads where applicable, and unlike Clark [20], our operator began without any prior conduction system pacing experience. The inclusion of CRT cases relatively early in the learning process also contributed to increased complexity. Nevertheless, the ultimate fluoroscopy times observed in our study (~5 min in the proficiency phase) are well aligned with benchmarks reported for complete LBBAP procedures in the literature, including the work of [15] (4–5 min in late-phase cases), [16] (8–12 min), and [23] (8–10 min), reflecting similar procedural efficiency once core technical skills are established. Therefore, our observations may be more representative of programs initiating LBBP without prior conduction-system pacing experience, where the learning curve encompasses not only lead placement but also the integration of all procedural steps required for complete device implantation.

The application of cumulative sum (CUSUM) analysis provided an objective and dynamic characterization of the learning curve. CUSUM is increasingly recognized as a modern performance-monitoring tool in cardiovascular surgery and interventional cardiology, enabling real-time identification of the transition from skill acquisition to procedural proficiency. These findings are consistent with prior reports in coronary artery bypass graft surgery and minimally invasive valvular interventions training, where CUSUM has been used to quantify operator learning trajectories and define competency thresholds [24,25,26]. By incorporating CUSUM into operator performance assessment, our study provides measurable proficiency benchmarks that can support structured training pathways and facilitate the safe and standardized implementation of LBBAP. To our knowledge, this is the first study to apply CUSUM analysis specifically to LBBAP, offering an objective complement to prior descriptive evaluations of procedural learning.

An important finding is that electrical performance and patient safety were maintained even during the early learning phase. Over 90% of patients achieved a pRWPT ≤ 75 ms, which is consistent with the 84.6% rate of confirmed LBB capture based on EHRA criteria pacing; the thresholds remained low, and no major complications occurred during implantation or follow-up. These results suggest that with careful technique and backup strategies, LBBAP can be safely implemented from the outset, even if procedural efficiency requires a longer maturation phase. Furthermore, our findings also suggest that CUSUM analysis may serve as a practical tool for monitoring performance and providing feedback in early-career operators, offering an objective framework to support structured skill acquisition and competency-based training in conduction system pacing.

### 4.1. Clinical Implications

In this single-operator experience, stable fluoroscopy performance was achieved after approximately 60–70 procedures. These values should be interpreted as observational findings rather than formal training requirements, as proficiency thresholds may vary across operators and institutions. Although this number is higher than some previously reported estimates, it may offer a realistic benchmark for centers implementing LBBAP de novo. Moreover, these findings support the incorporation of structured LBBAP training pathways in which novice operators begin with anatomically favorable non-CRT cases and progress in complexity under guided performance monitoring.

### 4.2. Limitations and Future Directions

Several limitations should be noted. First, we used total procedural fluoroscopy time as our primary efficiency metric. This approach captures the real-world complexity of complete device implantation, including backup right ventricular leads and atrial or defibrillator leads where applicable, but it does not isolate the fluoroscopy attributable solely to LBB lead deployment. Other learning-curve studies have focused specifically on ventricular lead fluoroscopy or on procedure time, which may yield shorter apparent learning curves but do not reflect the full implant workflow. Our results should therefore be interpreted as conservative estimates of the case volume required to achieve stable overall procedural efficiency in a de novo LBBP program. Second, the single-center, single-operator design limits generalizability but offers a clean view of one operator’s learning trajectory without contamination from multi-operator variability. In addition, although LBB capture success was quantified separately using EHRA criteria, the learning-curve analysis was intentionally performed on the full LBBAP cohort because the procedural workflow is identical across LBBP and DSP until the moment of electrophysiological confirmation.

Total procedure time was not systematically captured in the procedural records of this early program and could therefore not be analyzed without risk of incomplete data bias. Future studies should incorporate standardized documentation of procedure duration as an additional efficiency metric.

Lastly, the number of CRT procedures was small (*n* = 9), and the study was therefore not powered to define a CRT-specific learning curve; these observations should be interpreted cautiously.

Future multicenter prospective studies with standardized efficiency metrics and longer follow-up will be needed. Simulation-based training, advanced imaging, or integration of 3D mapping could potentially shorten the learning trajectory.

## 5. Conclusions

In conclusion, this study demonstrates that LBBAP can be safely introduced by operators without prior experience in conduction system pacing, with a high rate of EHRA-defined left bundle branch capture (84.6%), while deep septal pacing accounted for 15.4% of cases. In this cohort, procedural proficiency was achieved after approximately 65 non-CRT implants, after which fluoroscopy times stabilized, and technical performance became reproducible. However, this case number should not be viewed as a universal proficiency threshold. CUSUM analysis allowed this transition point to be identified objectively and in real time, rather than retrospectively. These findings support the use of CUSUM as a practical framework for monitoring operator progression, defining proficiency thresholds, and guiding competency-based training and credentialing pathways for centers adopting LBBAP.

## Figures and Tables

**Table 1:** Baseline clinical and demographic characteristics.

Variable	Value/%
Age	71.86 ± 7.33
Sex	Male: 68 (65.4%)
Dual chamber pacemaker (DDD)	82 (78.8%)
Single chamber pacemaker (VVI)	13 (12.5%)
Cardiac Resynchronization Therapy (CRT)	9 (8.7%)
AV blocks	74
Sick sinus syndrome	21
Cardiac resynchronization therapy indication	9
Heart failure with reduced EF (HFrEF)	23/104 (22.1%)
Dilated cardiomyopathy (DCM)	12/104 (11.5%)
Coronary artery disease (CAD)	17/104 (16.3%)
Diabetes mellitus	37/104 (35.6%)
Chronic kidney disease (CKD)	28/104 (26.9%)
Atrial fibrillation (AF)	32/104 (30.8%)
Pulmonary hypertension (PH)	20/104 (19.2%)
Peripheral arterial disease (PAD)	8/104 (7.7%)
Dyslipidemia	73/104 (70.2%)
Obesity	33/104 (31.7%)

Abbreviations: EF—ejection fraction; HFrEF—heart failure with reduced ejection fraction; DCM—dilated cardiomyopathy; CAD—coronary artery disease; CKD—chronic kidney disease; AF—atrial fibrillation; PH—pulmonary hypertension; PAD—peripheral arterial disease.

**Table 2:** Electrical parameters during implantation and follow-up.

Parameter	Implantation (Mean ± SD	1 Month (Mean ± SD)	3 Months (Mean ± SD)	Implantation Median (IQR/Range)	1 Month Median (IQR/Range)	3 Months Median (IQR/Range)
Pacing threshold (V @ 0.4 ms)	0.82 ± 0.22	0.42 ± 0.20	0.47 ± 0.18	0.90 (0.70–0.90)	0.50 (0.25–0.50)	0.50 (0.44–0.50)
R-wave sensing (mV)	17.8 ± 6.8	15.0 ± 5.7	9.96 ± 3.42	17.0 (12.7–23.5)	15.0 (12.0–15.0)	11.2 (10.7–11.6)
Impedance (Ω)	794.5 ± 170.1	527.3 ± 83.4	510.53 ± 81.18	776.5 (683.8–888.0)	511.0 (471.5–583.5)	494.5 (428.0–555.0)
RWPT in V6 (ms)	70.7 ± 8.2	–	–	–	–	–
Unipolar paced COI (mV)	19.2 ± 5.4	–	–	19.7 (14.5–24.0)	–	–
Unipolar sensed COI (mV)	11.9 ± 3.2	–	–	10.0 (10.0–14.0)	–	–

Abbreviations: V—volts; mV—millivolts; Ω—ohms; ms—milliseconds; SD—standard deviation; IQR—interquartile range; COI—current of injury; RWPT—R-wave peak time.

## Data Availability

The raw data supporting the conclusions of this article will be made available by the authors on request.

## Abbreviations

The following abbreviations are used in this manuscript:

Abbreviation	Definition
AF	atrial fibrillation
CAD	coronary artery disease
CKD	chronic kidney disease
COI	current of injury
CRT	cardiac resynchronization therapy
DCM	dilated cardiomyopathy
EF	ejection fraction
HFrEF	heart failure with reduced ejection fraction
IQR	interquartile range
LBB	left bundle branch
LBBP	left bundle branch pacing
PAD	peripheral arterial disease
PH	pulmonary hypertension
pRWPT6	paced R-wave peak time in lead V6
RWPT	R-wave peak time
SD	standard deviation
V	volts
mV	millivolts
Ω	ohms
ms	milliseconds
